# Local delivery of CpG-B and GM-CSF induces concerted activation of effector and regulatory T cells in the human melanoma sentinel lymph node

**DOI:** 10.1007/s00262-016-1811-z

**Published:** 2016-03-02

**Authors:** Mari F. C. M. van den Hout, Berbel J. R. Sluijter, Saskia J. A. M. Santegoets, Paul A. M. van Leeuwen, M. Petrousjka van den Tol, Alfons J. M. van den Eertwegh, Rik J. Scheper, Tanja D. de Gruijl

**Affiliations:** Department of Pathology, Cancer Center Amsterdam, Vrije Universiteit (VU) University Medical Center, De Boelelaan 1117, 1081 HV Amsterdam, The Netherlands; Department of Medical Oncology, Cancer Center Amsterdam, Vrije Universiteit (VU) University Medical Center, De Boelelaan 1117, Room CCA 2.44, 1081 HV Amsterdam, The Netherlands; Department of Surgical Oncology, Cancer Center Amsterdam, Vrije Universiteit (VU) University Medical Center, De Boelelaan 1117, 1081 HV Amsterdam, The Netherlands

**Keywords:** Immunotherapy, Melanoma, Sentinel lymph node, CpG, GM-CSF, Regulatory T cells

## Abstract

Impaired immune effector functions in the melanoma sentinel lymph node (SLN) may allow for early metastatic events. In an effort to determine the optimal way to strengthen immune defenses, 28 clinical stage I–II melanoma patients were randomized in a 3-arm Phase II study to receive, prior to excision and sampling of the SLN, i.d. injections of saline or low-dose CpG-B (CpG), alone or combined with GM-CSF (GM), around the melanoma excision site. We previously described the combined administration of these DC-targeting agents to result in activation and recruitment of potentially cross-presenting BDCA3^+^ DCs to the SLN. In this report we describe the effects on effector and regulatory T and NK cell subsets. Local low-dose CpG administration resulted in lower CD4/CD8 ratios, Th1 skewing, increased frequencies of melanoma-specific CD8^+^ T cells and possible recruitment of effector NK cells, irrespective of GM co-administration. These immune-potentiating effects were counterbalanced by increased IL-10 production by T cells and significantly higher levels of FoxP3 and CTLA4 in regulatory T cells (Tregs) with correspondingly higher suppressive activity in the SLN. Notably, CpG ± GM-administered patients showed significantly lower numbers of SLN metastases (saline: 4/9, CpG + GM: 1/9, CpG: 0/10, *p* = 0.04). These findings indicate that i.d. delivery of low-dose CpG ± GM potentially arms the SLN of early-stage melanoma patients against metastatic spread, but that antitumor efficacy may be further boosted by counteracting the collateral activation of Tregs.

**Electronic supplementary material:**

The online version of this article (doi:10.1007/s00262-016-1811-z) contains supplementary material, which is available to authorized users.

## Introduction

Cutaneous melanoma is the most aggressive type of skin cancer, and its incidence is increasing worldwide. Once melanoma spreads beyond its primary site, prognosis is very poor [1]. Therefore, in early-stage melanoma patients, an effective adjuvant treatment to counter metastatic spread would be highly valuable. Melanoma is also one of the most immunogenic tumors. Tumor-reactive T cells are already detectable in the blood and TDLN in early stages of tumor development [2]. In recent years, it has become increasingly clear that melanoma evades and suppresses the immune system, thereby precluding an effective antitumor response and hampering the effect of cancer immunotherapy [3, 4]. As professional APCs and prime orchestrators of the innate and adaptive immune response, dendritic cells are obvious targets of melanoma-induced immune suppression [5]. When suppressed, they prevent an effective antitumor immune response and promote tolerance to melanoma-associated antigens (MAA). The first-line TDLN, the sentinel lymph node (SLN), takes the brunt of the melanoma-associated immune suppression, making it in effect an “immune-privileged site” with the ability to assert and maintain a state of systemic tolerance to MAA [3, 6]. Impaired immune effector functions in the SLN most likely contribute to the early metastatic events that are associated with melanoma.

The sentinel lymph node procedure (SNP) presents a unique translational setting to study adjuvant immune therapies in vivo [7]. To assess the functional immune status of the SLN, we developed a previously reported sampling technique whereby viable immune effector cells can be obtained without interfering with standardized diagnostic procedures [8]. By scraping the surface of bisected SLN, flow cytometric analysis of myeloid and lymphocytic subsets and expansion of functional T cells can be achieved, while allowing regular lengthwise lamellar sampling of the whole SLN for immunohistochemical assessment of tumor involvement. The feasibility and non-interference with diagnostic outcome were subsequently confirmed by others [9].

In previous work we investigated the applicability of i.d. administration of GM-CSF or CpG in early-stage melanoma patients to reverse melanoma-induced immune suppression and mount an effective antitumor response. Both the targeting of conventional myeloid DCs by GM-CSF and plasmacytoid DCs (pDCs) by CpG elicited impressive activation, maturation and recruitment of DCs to the SLN and a subsequent increase in melanoma-specific CD8^+^ T cells, supporting the utility of these agents as adjuvant treatment in early-stage melanoma patients [10–13]. Since both compounds have been shown to interact synergistically to induce an antigen-specific antitumor immune response in murine models [14, 15] and proved to be clinically safe [16], we combined CpG and GM-CSF, both at lower doses than before to minimize side effects (1 mg and 100 μg, versus 8 mg and 3 µg/kg/day for 4 consecutive days, respectively), and studied their effects in a randomized Phase II trial in comparison with administration of low-dose CpG alone or saline.

As recently reported by us [17], this combined administration resulted in activation of the full range of DC subsets and recruitment of potentially cross-presenting BDCA3^+^ DCs to the SLN. Here we report on the subsequent effects on the T and NK effector cells. Overall, our data point to dominant effects of CpG over GM-CSF at the administered doses. Beside lowering CD4/CD8 ratios, skewing to a type-1 T cell response profile, possibly inducing NK effector trafficking and increasing MAA-specific CD8^+^ T cell frequencies, we show that CpG has clear collateral suppressive effects in vivo, specifically by activating regulatory T cells (Tregs).

## Materials and methods

### Patients, clinical procedures, and cell sampling

From September 2006 until May 2008, 28 patients were included in this single-blinded Phase II study and randomly assigned to receive preoperative local administration of either GM-CSF (Leukine, Berlex Laboratories Inc. Montville, NJ) and synthetic CpG-B (PF-3512676, Coley Pharmaceutical Group, Inc., Wellesley, MA), CpG-B alone or saline (NaCl 0.9 %). All patients were clinically diagnosed with stage I/II melanoma, according to criteria of the American Joint Committee on Cancer, and were scheduled to undergo SNP. Inclusion criteria were as described previously [13]. The Institutional Review Board of the VU University Medical Center approved the study, and written informed consent was obtained from each patient prior to treatment.

All patients received 4 ml i.d. injections directly into the scar of the primary melanoma excision, 7 and 2 days before SNP; injections consisted of either a combination of 1 mg CpG and 100 μg of GM-CSF, 1 mg CpG or 4 ml plain saline. Heparinized blood was drawn prior to the first injection (*t* = −7) and on the day of the SNP (*t* = 0). Viable PBMCs were isolated and cryopreserved for further analysis as previously described [12]. One week after the first injection all patients underwent SNP and reexcision of the primary melanoma site as described [18]. SLNs were collected in sterile ice-cold IMDM supplemented with 25 mM HEPES buffer (BioWhittaker, Verviers, Belgium) with 10 % FCS, 50 IU/ml penicillin–streptomycin, 1.6 mM l-glutamine and 0.05 mM β-mercaptoethanol [i.e., complete medium (CM)]. Viable cells were scraped from the SLN using a previously described method [8]. SLN cells were washed twice in CM, counted, and further processed.

### T cell expansion

T cells from all SLN were expanded as described previously [11]. Briefly, cells were incubated for 1 h on ice with 2 µg anti-CD3 and 0.4 µg anti-CD28 per 10^6^ cells (clones 16A9 and 15E8, kindly provided by Dr. René van Lier, Sanquin, Amsterdam, the Netherlands) in 100–200 µl CM with 5 % FCS. After incubation and washing, cells were placed in 24-well plates (Greiner Bio-One) pre-coated with affinity-purified goat antimouse Ig (1:100; DAKO) in CM with 10 % FCS at a concentration of 10^6^/ml/well for 1 h at 4 °C. Cells were subsequently cultured for 48 h in a humidified 5 % CO_2_ incubator at 37 °C. After 24 h 100 μl supernatant from each well was stored for T cell cytokine analysis (see next paragraph). After 48 h, cells were resuspended and the contents of each well were divided over four new uncoated wells at 250 µl/well. To each well, 750 µl CM supplemented with 14 IU/ml recombinant human IL-2 (Sanquin) was added, resulting in a final concentration of 10 IU/ml rhIL-2. Cells were cultured for another 5 days, after which they were harvested and counted. All SLN T cells underwent two expansion cycles. Finally, the expanded T cells were harvested and cryostored for functional analysis at a later date.

### T cell cytokine profiling

Supernatants from the 24-h polyclonal T cell ex vivo expansion cultures were analyzed using the Type 1/2 T helper (Th1/Th2) Cytometric Bead Array kit for flow cytometric detection of IL-2, IL-4, IL-5, IL-10, TNF-α and IFN-γ, following the manufacturer’s instructions and using Cytometric Bead Array analysis software (BD Biosciences, San Jose, CA).

Expanded T cells were used for analysis of intracellular T cell cytokines. 1 × 10^6^ cells were stimulated for 4½ h in a humidified 5 % CO_2_ incubator at 37 °C with 50 ng/ml PMA and 500 ng/ml ionomycin in the presence of 1:500 Golgiplug (BD Biosciences) resuspended in Yssels medium at a concentration of 2 × 10^6^/ml. After stimulation cells were washed and stained.

### Flow cytometry

Freshly isolated SLN cells, expanded SLN T cells or thawed PBMCs were directly stained with antibodies labeled with either FITC, PE, PE-CY5.5, PerCP-CY5.5 or allophycocyanin and analyzed by flow cytometry at 100,000 or 200,000 events per measurement, as previously described [8, 9]. Monoclonal antibodies against CD3, CD4, CD8, CD56, CD19, CD25 (BD Biosciences) and latency-associated peptide (LAP) (R&D Systems Inc. Minneapolis, MN) with matching isotype control antibodies were used. Intracellular FoxP3 staining was done using the eBioscience (San Diego, CA) PE-antihuman FoxP3 staining set following the manufacturer’s instructions. CTLA4, IFN-γ, TNF-α, IL-2, IL-4 and IL-5 were stained intracellularly using the Cytofix/Cytoperm Kit with GolgiStop (BD Biosciences) as described [19]. LAP staining was performed as described [20]; CD4- and CD25-enriched and CD4- and CD25-expanded T cells were stimulated for 48 h with anti-CD3 and anti-CD28 mAbs (both at 1 μg/ml) in the presence of 10 IU IL-2 after which they were stained according to the published protocol [20].

### Suppression assay

Tregs were enriched from expanded SLN T cells and PBMC. After resuspending cells in PBS + 0.5 %BSA + EDTA an untouched CD4 MACS isolation was performed according to the manufacturer’s protocol (Miltenyi Biotec, Bergisch Gladbach, Germany), routinely achieving purities of approximately 95 %. In addition, a CD25 magnetic bead-mediated isolation was performed twice to select for CD4^+^CD25^hi^ cells (Miltenyi). Average Treg purity after enrichment, defined as % CD3^+^CD4^+^CD25^hi^FoxP3^+^ cells of total T cells, was 32.9 ± 4 and 43.9 ± 3.1 % from expanded SLN T cells and PBMC, respectively. Purity did not differ significantly between the three groups (*p* = 0.62 and *p* = 0.54 for SLN and PBMC, respectively). Enriched Tregs were resuspended in CM at 0.5 × 10e6/ml.

Effector cells were isolated from two different buffy coats. After untouched CD4 isolation, CD25^+^ cells were depleted using CD25 magnetic beads according to the manufacturer’s protocol (Miltenyi). Until use in suppression assays, CD4^+^CD25^−/low^ cells were cryostored. After thawing, CD4^+^CD25^−/low^ effector cells were resuspended in PBS + 0.1 % BSA at 1 × 10e6 cells/ml and incubated for 7 min at 37 °C with 3 µM carboxyfluorescein succinimidyl ester (CFSE). Cells were washed and resuspended in CM for 15 min to stabilize CFSE staining. After a final wash step, cells were resuspended in CM at 0.5 × 10e6/ml as previously described [21].

5 × 10^4^ Tregs were cultured at a 1:1 ratio with allogeneic CD4^+^CD25^−/low^ cells. The cells were stimulated with anti-CD3 mAb (1 μg/ml) and anti-CD28 mAb (1 μg/ml) in the presence of 10 IU IL-2 in 96-well round-bottom plates. After 3 days, proliferation (i.e., CFSE dilution) of responder cells was analyzed by FACS. Assays were performed in triplicate or duplicate, and suppression was calculated as the average decrease of proliferation of effector cells compared to positive controls (i.e., effector cells without added Tregs).

### Tetramer staining

PE-labeled HLA-A2 tetramers (kindly provided by Dr. Ton Schumacher, Netherlands Cancer Institute, Amsterdam, the Netherlands) presenting melanoma-associated epitopes Gp100_154–162_, Gp100_209–217_, Gp100_280–288_, MAGE-A3_271–279_, MART-1_26–35_, NY-ESO_157–165_ and TYR_369–377_ were used to detect MAA-specific CD8^+^ T cells. Gp100 tetramers were pooled. Tetramer staining was performed as previously described [22].

### Statistics

Overall differences between the three patient groups in terms of patient and SLN characteristics and immune parameters were analyzed using the Kruskal–Wallis test. The post hoc multiple comparison Dunn’s test was used to analyze differences between two patient study groups. For differences between two groups the Mann–Whitney test was used. Correlations were determined using the Pearson r test. Differences were considered statistically significant when *p* < 0.05.

## Results

### Patient characteristics

A total of 28 patients with clinical stage I–II melanoma were included in this study. Patients were randomly assigned to one of three test groups: receiving two i.d. injections of (1) saline, (2) CpG-B (1 mg) and GM-CSF (100 μg) (CpG + GM) or 3) CpG-B only (1 mg) (CpG). Injections were administered 7 and 2 days prior to SNP. Adverse effects of CpG ± GM were transient and mild flu-like symptoms [17]. After pathological examination, 5 of 28 SLNs contained tumor cells, corresponding to pathologically confirmed stage III disease. Four of these stage III patients had received saline, resulting in a remarkable and statistically significant difference in lymph node positivity between groups (*p* = 0.04). Of these lymph node metastases, two had a diameter greater than 2 mm, one in the saline and the other in the CpG + GM group. All patients but one with a positive SLN underwent an additional lymph node dissection, and all additional nodes were found to be tumor negative. The one patient for whom a watchful waiting policy was followed harbored a micrometastasis, but developed a clinically detectable regional lymph node metastasis 8 months after SNP, whereupon a delayed completion lymph node dissection was performed. HLA-A2 status was determined by flow cytometry to enable testing of specific CD8^+^ T cell frequency for a panel of melanoma-derived CD8^+^ T cell epitopes. Patient characteristics are summarized in Table 1.

**Table 1:** Patient and sentinel lymph node characteristics

	Saline	CpG + GM	CpG	*p* ^†^
Gender (male:female)	4:5	4:5	6:4	0.74
Age (mean ± SD)	47 ± 12	50 ± 12	58 ± 12	0.16
Breslow thickness [mean ± SD (mm)]	1.67 ± 0.55	2.19 ± 1.37	1.73 ± 0.96	0.67
Tumor-positive SLNs	4/9	1/9	0/10	**0.04**
Additional lymph node dissection	3/9	1/9	0/10	0.12
Time from primary excision to SNP [mean ± SD (days)]	45 ± 19	47 ± 9	47 ± 20	0.77
HLA-A2	4/9	5/9	8/10	0.28

^†^By Kruskal–Wallis test

Bold value indicates statistical difference

### Lymphocyte subsets and T cell cytokine profiles: CD4/CD8 shift and Th1 skewing

No significant differences in frequencies of B and NK cells from freshly isolated SLN cells were found between patient groups. However, in both CpG test groups we observed significantly decreased frequencies of CD4^+^ T cells leading to significantly lower CD4/CD8 ratios in the CpG + GM group. The expression levels of the activation markers CD25, CTLA4 and FoxP3 in activated CD4^+^ T effector cells (Tact, i.e., with intermediate CD25 and FoxP3 expression levels) in the SLN were slightly, but not significantly, higher in the CpG test groups (Table 2).

**Table 2:** Sentinel lymph node lymphocyte subset frequencies

	Lymphocyte subsets^a^					CD4:CD8 ratio	Tact^b^ CD25^+^ FoxP3^int^ (%)	Treg^b^ CD25^hi^ FoxP3^hi^ (%)	Treg:Tact ratio
	B cells (%)	NK cells (%)	T cells (%)	CD4^+^ T cells (%)	CD8^+^ T cells (%)				
Saline	14.2	1.25	77.9	66.5	8.8	8.9	3.87	5.41	2.30
Range	9.9–20	0.7–2.2	69–85	55–75	3.5–12	5.1–20	1.3–9.0	2.3–9.5	0.3–4.4
CpG + GM	16.7	1.56	70.9	51.8*	15.1	3.9*	6.36	5.60	1.08
Range	11–28	1.1–2.0	51–80	32–67	9.0–22	2.1–6.6	2.2–9.1	2.9–11	0.6–2.4
CpG	17.7	1.38	68.1*	52.5*	10.9	5.6	4.44	5.36	1.60
Range	6.9–35	0.7–2.2	52–78	36–60	5.2–16	2.4–10	1.6–8.0	2.7–8.0	0.4–3.3

* *p* < 0.05 versus saline by Kruskal–Wallis test with Dunn’s post-test

^a^Indicated percentages are of measured SLN leukocytes

^b^Indicated percentages are of CD3^+^CD4^+^CD25^int^ T cells

Bold values indicates statistical differences

**Fig. 1:**
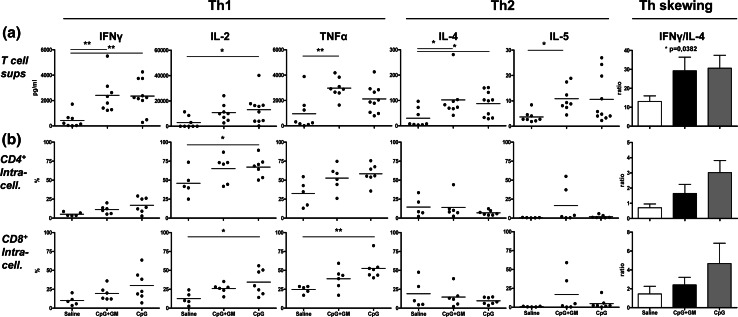
T cell cytokine profiles reveal CpG-associated Th1 skewing. **a** Cytokine secretion profiles and Th skewing (represented as the average of all IFN-γ/IL-4 ratios) of freshly harvested SLN T cells after in vitro stimulation. Means (in *bar graphs* with SEM) are shown. **b** Intracellular cytokine levels in expanded SLN CD4^+^ (*top row*) and CD8^+^ (*bottom row*) T cells after in vitro stimulation. Shown are percentages of cells positive for the indicated intracellular Th1 and Th2 cytokines and Th skewing. *p < 0.05; **p < 0.01

To determine the effects of local CpG or CpG + GM administration on T cell skewing in the melanoma SLN, we measured Th1 and Th2 cytokines in supernatants of freshly isolated SLN cells ex vivo, which were stimulated overnight by immobilized anti-CD3 and anti-CD28. No clear differences were observed between the CpG and CpG + GM groups. However, compared to the saline group, CpG ± GM administration led to increased production of all measured cytokines and resulted in significantly skewed Th1 profiles as determined by IFN-γ/IL-4 ratios (Fig. 1a).

A statistically nonsignificant trend was observed for the association of type-1 skewed profiles with CpG ± GM administration in CD4^+^ and CD8^+^ T cells as detected by intracellular cytokine expression *after* expansion (Fig. 1b). There was a general lack of detectable type-2 cytokine expression. Although consistent with the considerably (10- to 100-fold) lower concentrations of released Th2 cytokines in pre-expansion populations (as shown in Fig. 1a), this might also have resulted from the expansion procedure.

### CpG/GM effects on NK cells

**Fig. 2:**
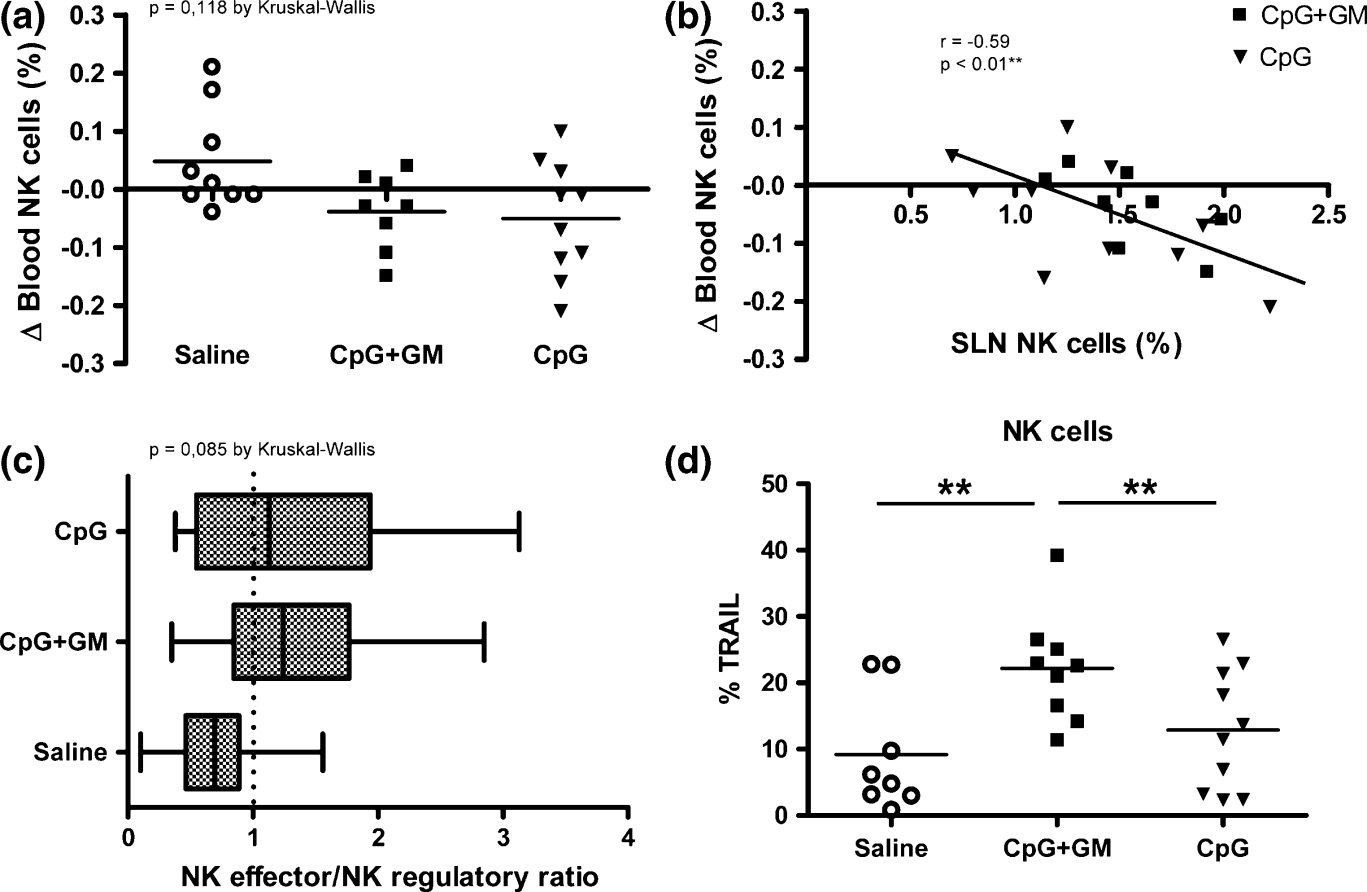
CpG/GM effects on NK cells. **a** Absolute changes in percentages of blood NK cells between *t* = −7 (pre-treatment) and *t* = 0 (post-treatment) are shown. **b** Changes in peripheral blood NK cell frequencies for CpG ± GM-administered patients are significantly and reversely correlated to corresponding SLN NK cell frequencies. **c** Shift from predominant regulatory CD56^bright^ to more effector CD56^dim^ NK cells in the melanoma SLN. Average CD56^dim^/CD56^bright^ ratios are shown for each group by *box* and *whisker plot*. **d** Surface TRAIL expression on SLN NK cells. *p < 0.05; **p < 0.01

In contrast to saline administration, after CpG as well as CpG + GM administration NK cell frequencies in the peripheral blood on average decreased (Fig. 2a). Although this difference was limited and did not reach statistical significance, the changes in NK cell frequencies in the peripheral blood correlated significantly (*p* < 0.01) with frequencies of NK cells in SLNs of CpG ± GM-administered patients, suggesting a recruitment of NK cells to the SLN upon CpG ± GM administration (Fig. 2b). Although not statistically significant (*p* = 0.085), we observed a shift from a predominant CD56^bright^ regulatory SLN NK cell population in the saline-administered group toward more CD56^dim^ effector SLN NK cells in both CpG test groups (Fig. 2c). In the CpG + GM group, we found highly significantly (*p* < 0.01) elevated percentages of NK cells with surface expression of TRAIL compared to the saline as well as the CpG group (Fig. 2d), pointing to an essential role for GM-CSF in this up-regulation. Of note, the same held true for TRAIL expression on SLN T and B cells (data not shown).

### Increased Treg activation in the SLN, but not in peripheral blood

**Fig. 3:**
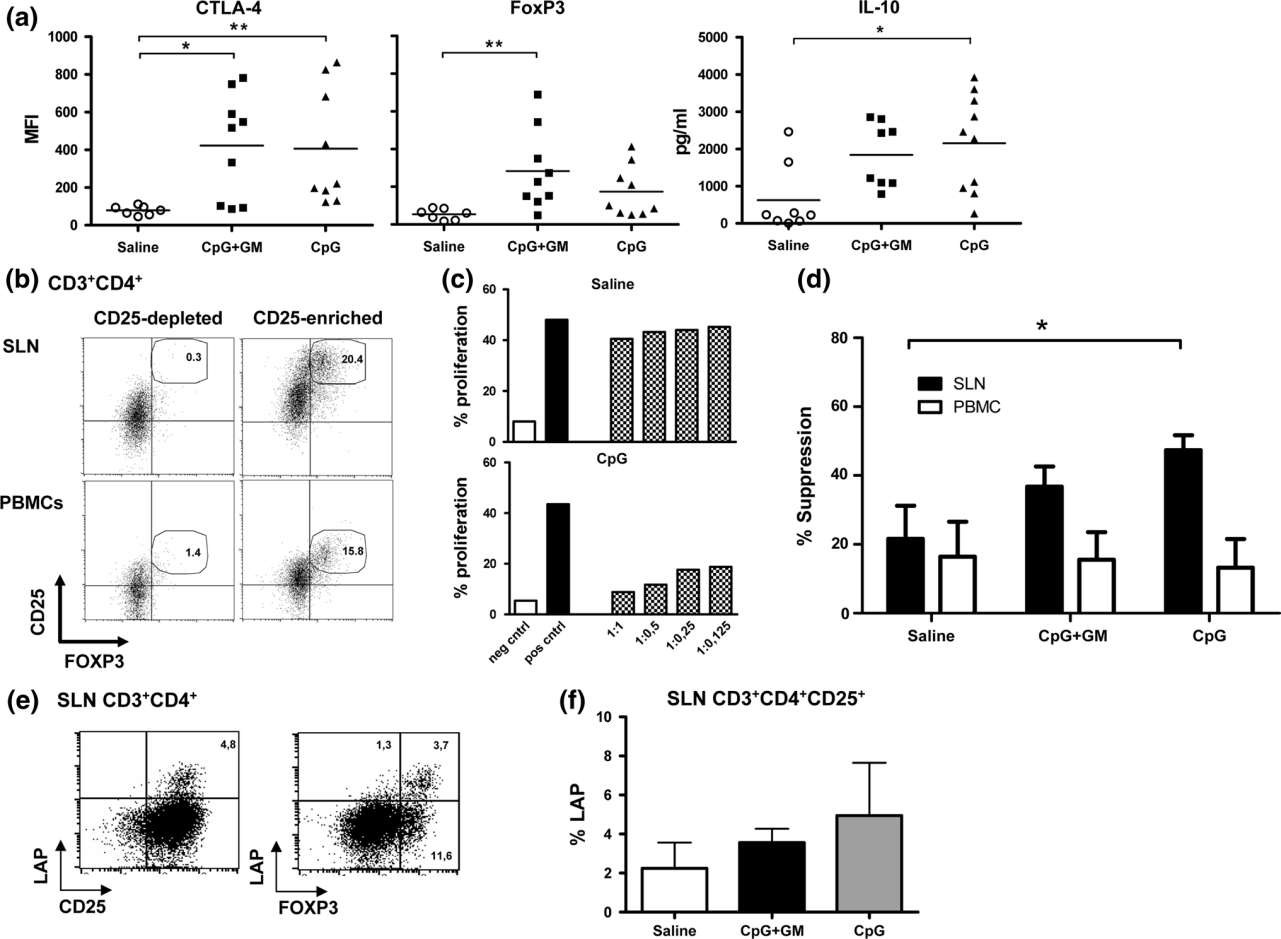
Increased Treg activation and suppressive activity in SLN, but not in peripheral blood. **a** CTLA-4 and FoxP3 expression levels [by mean fluorescence intensity (MFI)] in freshly isolated SLN Tregs and IL-10 secretion after stimulation of freshly harvested SLN T cells. *p < 0.05; **p < 0.01. **b** Representative Treg staining of CD25-depleted and CD25-enriched expanded SLN T cells and of T cells from the peripheral blood,  % Tregs are listed. **c** Representative suppression assays from a saline- and a CpG-administered patient. *Gray bars* show the percentage of proliferated CD4^+^CD25^−^ effector cells in the presence of CD4^+^CD25^+^-enriched fractions of expanded SLN T cells at different ratios. **d** *Closed bars* indicate the suppressive activity of CD4/CD25-enriched, expanded SLN T cells for all three groups. *Open bars* indicate the suppressive activity of CD4/CD25-enriched T cells from the peripheral blood at the same time point as SLN harvest (*t* = 0). Numbers of patients tested in the SLN and peripheral blood are, respectively: saline: 5/6, CpG + GM: 7/6, CpG: 6/5. **e** Representative CD25/LAP and FoxP3/LAP staining after pre-gating on CD3^+^CD4^+^ cells. **f** LAP expression of CD3^+^CD4^+^CD25^+^ cells from expanded SLN T cells. Average percentages of LAP with SEM are shown for each group. *N* = 4 in each group

Neither in freshly isolated SLN cells nor in the peripheral blood, differences in percentages of natural Tregs (nTregs, defined as CD3^+^CD4^+^CD25^hi^FoxP3^+^) were observed between patient groups (Table 2; data not shown for peripheral blood). There was, however, a clear and significant difference in levels of expression of FoxP3 and CTLA-4 in freshly isolated SLN Tregs between both CpG test groups and the saline control group (Fig. 3a). In contrast, FoxP3 levels in Tregs isolated from peripheral blood (*t* = 0) were comparable between groups (MFI, saline: 197 ± 14, CpG + GM: 202 ± 126, CpG: 160 ± 75). In addition, in supernatants of freshly isolated SLN cells from both CpG test groups, we found elevated levels of IL-10 after overnight stimulation by immobilized anti-CD3 and anti-CD28 compared to the saline control group, reaching significance for the CpG group (Fig. 3a). To assess suppressive functionality of Tregs, enriched fractions from PBMC and expanded SLN T cells were used. To obtain sufficient cells for functional testing of Treg activity we were forced to expand SLN T cells. Figure 3b shows representative Treg (CD25/FoxP3) staining after magnetic bead-mediated Treg enrichment from SLN and PBMC samples. Generally high CD25 levels in SLN Tregs resulted from their expansion and concomitant activation. Two representative suppression assays, one from a saline and the other from a CpG-administered patient, are shown in Fig. 3c. For comparison between groups we used the relative suppression of proliferation of CD4^+^CD25^−^ target cells by CD4^+^CD25^+^-enriched fractions from expanded SLN T cells and PBMC at a 1:1 ratio. Corresponding to the elevated IL-10 release and high expression levels of suppressive FoxP3 and CTLA-4 in unexpanded Tregs from the SLN we observed a statistically nonsignificant trend (*p* = 0.09) toward increased suppressive activity of Treg-enriched fractions from expanded SLN T cells in CpG-administered patient groups (Fig. 3d). In contrast, no difference in suppressive activity of Treg-enriched populations from peripheral blood was observed between patient groups, in keeping with the equivalent FoxP3 expression levels in these cells (Fig. 3d).

LAP is expressed on the cell surface of activated, but not resting Tregs, and has not only been shown to be useful in the purification of Tregs from expansion cultures, but also as a marker of Tregs for immune-monitoring studies in patients treated with active immunotherapy [20, 23]. We stained extracellular LAP after 48 h of anti-CD3/anti-CD28-mediated stimulation of Treg-enriched fractions of expanded SLN T cells as previously described [20]. Figure 3e shows LAP expression in relation to FoxP3 and CD25 from a representative patient. Indeed, LAP^+^ Tregs were also highly positive for CD25 and FoxP3, in keeping with their reported regulatory activity. We observed a statistically nonsignificant trend toward higher frequencies of LAP^+^ Tregs in the CpG and to a lesser extent in the CpG + GM group, as compared to the saline group (Fig. 3f), corresponding to the observed IL-10 release (Fig. 3a) and suppressive activity in these groups (Fig. 3d).

### Increased melanoma-specific CD8^+^ T cell frequencies in CpG + GM-treated SLN

**Fig. 4:**
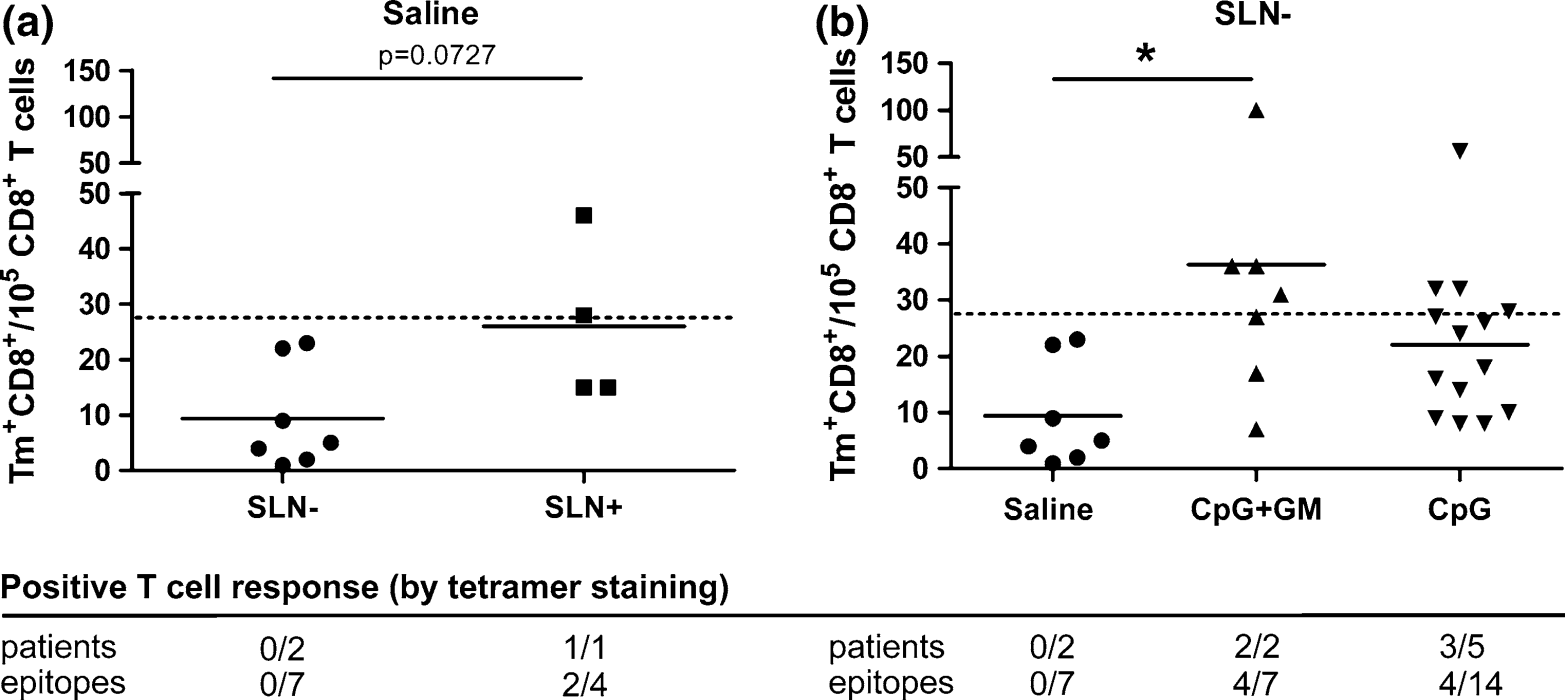
MAA-specific CD8^+^ T cells in the SLN. **a** MAA-specific tetramer^+^CD8^+^ T cell rates in the SLN of HLA-A2^+^ saline-administered patients are shown for tumor-negative and tumor-positive SLNs. Each dot represents one melanoma-specific tetramer-binding CD8^+^ population. The cutoff threshold set for positive tetramer responses is shown as a *dashed line*. **b** MAA-specific tetramer^+^CD8^+^ T cell rates in tumor-negative SLNs for all three groups. Below both graphs response numbers relative to evaluated numbers of patients and epitopes are shown. *p < 0.05

We determined CD8^+^ T cell frequencies against a panel of MAA by tetramer binding of HLA-A2^+^ patients with sufficient numbers of T cells expanded from the SLN suspensions (Fig. 4a). We stratified tetramer-binding results according to SLN tumor status because, in accordance with previous reports [2], a trend (*p* = 0.07) toward higher tetramer-binding rates was found in tumor-positive SLN from saline-administered patients (Fig. 4a). Consistent with our previous studies of CpG or GM-CSF single administration [11, 12], we found significantly higher levels of MAA-specific CD8^+^ T cell rates in tumor-negative SLNs of combined low-dose CpG and GM-CSF-administered patients compared to the tumor-negative control group (Fig. 4b). Low-dose CpG only also resulted in higher tetramer response rates, but this did not reach statistical significance.

A tetramer response was considered positive when the frequency of CD8^+^ tetramer-binding T cells exceeded the mean tetramer response rate plus two times the standard deviation in the tumor-negative control group (i.e., 28 tetramer^+^CD8^+^ T cells/10^5^ CD8^+^ T cells). Positive responses are listed in Fig. 4a, b, showing that five out of seven evaluable CpG ± GM-treated patients showed a positive response (8/21 epitopes tested), while neither of the two evaluable tumor-negative control patients showed a positive response (0/7 epitopes tested).

## Discussion

In previous studies of single i.d. administration of either GM-CSF or CpG, we found evidence for antimelanoma immune potentiation of the primary tumor site and its lymphatic drainage area through the boosting of tumor-specific CD8^+^ T cells [11, 12]. In the current study we monitored the effects of combined i.d. administration of low-dose GM-CSF and CpG and found a full-range activation of DC subsets in SLN (migratory and lymph node resident) and peripheral blood as well as a recruitment of BDCA3/CD141^+^ DC subsets with cross-priming ability to the SLN [17]. In the current report we have endeavored to assess the functional consequences of this adjuvant treatment in terms of effector and regulatory lymphocyte activation. Except for the up-regulation of TRAIL after CpG + GM administration on lymphocyte subsets and NK cells, we did not observe obvious differences between CpG and CpG + GM administration. This seems contradictory to the reported synergy between these two agents [14, 15] and may be explained by the small size of our study groups. This lack of power also calls for caution in the interpretation of some of our other results. The monitoring of melanoma-specific CD8^+^ T cells in particular was hampered by the limited number of cells and HLA-A2-positive patients evaluable. Nevertheless, in concordance with our previous studies, we found significantly increased frequencies of MAA-specific CD8^+^ T cells in the SLN of low-dose CpG + GM-administered patients [11, 12]. Moreover, the apparent dominant effect of CpG over GM-CSF in terms of T cell activation corresponds to our previous findings for DC recruitment and activation [17].

Others have combined CpG and GM-CSF with melanoma-associated peptides for vaccination in patients with advanced (stage III–IV) melanoma and have shown it to be a safe and promising strategy, but with poor to moderate clinical results, which were attributed to the presence of local regulatory responses and the exhausted phenotype of tumor infiltrating lymphocytes [16]. Although T cell exhaustion is believed to play a minor role in early-stage malignancies, regulatory responses may well be an important hurdle for effective immunotherapy in early-stage melanoma [24, 25]. Therefore, beside monitoring the effector arm of the immune system, we were interested in potentially suppressive effects of CpG and GM-CSF, particularly since recent evidence shows that CpG can induce IDO and STAT-3 expression in pDCs with collateral activation of Tregs [26–28]. In our previous study of single dose (8 mg) CpG administration we observed lower percentages of CD4^+^CD25^hi^ SLN Tregs in CpG-administered patients [10]. In the current study of low-dose CpG ± GM, we found no differences in SLN Treg frequencies between groups. This apparent discrepancy might be attributable to the difference in dosing and timing of CpG administration, to higher numbers of SLN samples included in the analyses or to more accurate Treg analyses used in the current study (using freshly harvested T cells and consistently including FoxP3 in the gating strategy).

More importantly, we found that intradermal CpG ± GM administration led to significantly higher expression levels of CTLA-4 and FoxP3 (CpG + GM group only) in freshly isolated SLN Tregs and to a correspondingly higher, but statistically nonsignificant, suppressive activity of Treg-enriched fractions from expanded SLN T cells. In contrast, no differences in Treg activation and suppressive activity were observed in peripheral blood. To obtain sufficient cells for functional testing of Treg activity we were forced to expand T cells from SLN. It has previously been shown that the suppressive potential of Tregs is preserved after in vitro anti-CD3/anti-CD28 expansion [29, 30]. Conversely, we found no evidence for an expansion-induced increase in suppression, since expanded Tregs from PBMCs showed similar suppressive activity as their unexpanded counterparts in two parallel tests we performed (Suppl. Fig. 1). The recent identification of LAP as a means to identify highly suppressive Tregs in expanded or activated T cell populations [20, 31, 32] prompted us to monitor its expression in our patients. The observed trend toward higher frequencies of LAP^+^ Tregs in expanded SLN T cells from CpG ± GM-administered patients might reflect their increased suppressive potential.

It cannot be excluded that in addition to nTregs, induced Tregs were involved in the increased suppressive activity of Tregs from CpG-conditioned SLNs. Induced Tregs are hard to distinguish from nTregs since no specific markers exist [33]. Their primary mode of action appears to be through the secretion of IL-10 (Tr1 cells, i.e., induced regulatory T cells) [34] or TGF-β (Th3 cells) [31]. An indication for the increased induction of Tregs after CpG administration is the significantly increased IL-10 release by freshly harvested T cells from the CpG-conditioned SLN. In addition, IFN-α strongly enhances IL-10-induced Tr1 differentiation [35]. Although CpG-B is a relatively weak IFN-α inducer, we have demonstrated that it induced a systemic transcriptional IFN-α response signature in our patients [17]. Finally, the significantly elevated levels of CTLA-4 in the SLN Tregs of CpG-administered patients could have contributed, via the proposed model of reverse signaling in pDCs, to the induction and activation of Tregs by IDO^+^ pDCs [36].

We observed a remarkably lower number of tumor-positive SLNs in the CpG ± GM-administered groups compared to saline-administered patients (Table 1). We interpret this to result from an effective antitumor immune response, boosted by the locally applied CpG ± GM. This may seem unlikely (i.e., the elimination within 1 week of metastases that persisted or grew out between primary tumor excision and SLN procedure), however, recent studies have demonstrated the ability of T cells, when properly unleashed, to rapidly clear even bulky tumors, and all metastases found in the SLN were clinically occult and often very small (<2 mm) [43]. As such, this observation would suggest that the increase in local suppressive markers after CpG ± GM administration does not outweigh the immune stimulatory properties of these agents. But, because this study was not powered to detect this unanticipated difference, it might be a mere coincidental finding. To exclude the possibility that the administration of CpG ± GM or the scraping procedure led to false-negative SLNs, we are closely monitoring the follow-up of all patients and, encouragingly, preliminary data point to prolonged recurrence-free survival of the intervention groups (Koster, van den Hout and colleagues, manuscript in preparation).

Because of the imbalance in SLN positivity, comparisons between groups at an immunological level might be biased. To make sure the observed immunological differences were not related to the presence of tumor cells we compared the immunological results from the CpG ± GM-administered patients to SLN-positive and SLN-negative patients from the saline group (see Suppl. Fig. 2 for representative examples). This clearly demonstrated that the immune stimulatory and regulatory effects on a T and NK cell level were related to CpG ± GM administration and not to the presence of tumor cells.

Our finding that CpG ± GM administration in melanoma patients enforces antitumor immunity through DC activation, a skewed Th1 response, and the boosting of CD8^+^ cytotoxic T cell responses is in keeping with previous observations in animal studies [14, 37]. Beside T cell immunity, an important role of NK cells, both in direct antitumor immunity and through shaping of the adaptive immune response via DC activation and editing, is becoming increasingly clear [38]. In murine models, tumor rejection after peritumoral injection of CpG was found to be dependent on NK cell recruitment to the tumor site, which led to the induction of a tumor-specific CTL response via cross-presentation by DCs [39, 40]. Some corroborating evidence is found in human studies: a phase II trial with subcutaneous CpG in metastatic melanoma patients showed that clinical benefit was associated only with NK cell cytotoxicity [41]. In the present study we found circumstantial evidence for a CpG ± GM-induced recruitment of NK cells to the SLN, corresponding to murine studies in which more conclusive evidence for TLR-mediated NK cell recruitment could be obtained [40, 42]. Together with a trend toward more effector CD56^dim^ NK cells after CpG ± GM administration and increased expression of TRAIL after CpG + GM, this points to increased tumoricidal activity of SLN NK cells.

Overall, we conclude that, although CpG ± GM-administered patients showed significantly lower numbers of SLN metastases, additional measures to minimize loco-regional Treg activity might optimize the clinical efficacy of CpG and GM-CSF as immune-potentiating agents for early-stage melanoma patients.

## Electronic supplementary material

Below is the link to the electronic supplementary material.

Supplementary material 1 (PDF 115 kb)

## Abbreviations

BDCA3: Blood dendritic cell antigen 3
CFSE: Carboxyfluorescein diacetate succinimidyl ester
CM: Complete medium
LAP: Latency-associated peptide
MAA: Melanoma-associated antigens
pDC: Plasmacytoid DC
SLN: Sentinel lymph node
SNP: Sentinel lymph node procedure
Tact: Activated T cells
Th1/Th2: Type 1/2 T helper
Tregs: Regulatory T cells
