# Supplementary material for: Local delivery of CpG-B and GM-CSF induces concerted activation of effector and regulatory T cells in the human melanoma sentinel lymph node
Source: Cancer Immunol Immunother. 2016 Mar 2;65(4):405–15. doi: 10.1007/s00262-016-1811-z (PMC4826413; doi:10.1007/s00262-016-1811-z)
Supplement: Supplementary file 1 — Supplementary material 1 (PDF 115 kb) [file 262_2016_1811_MOESM1_ESM.pdf]

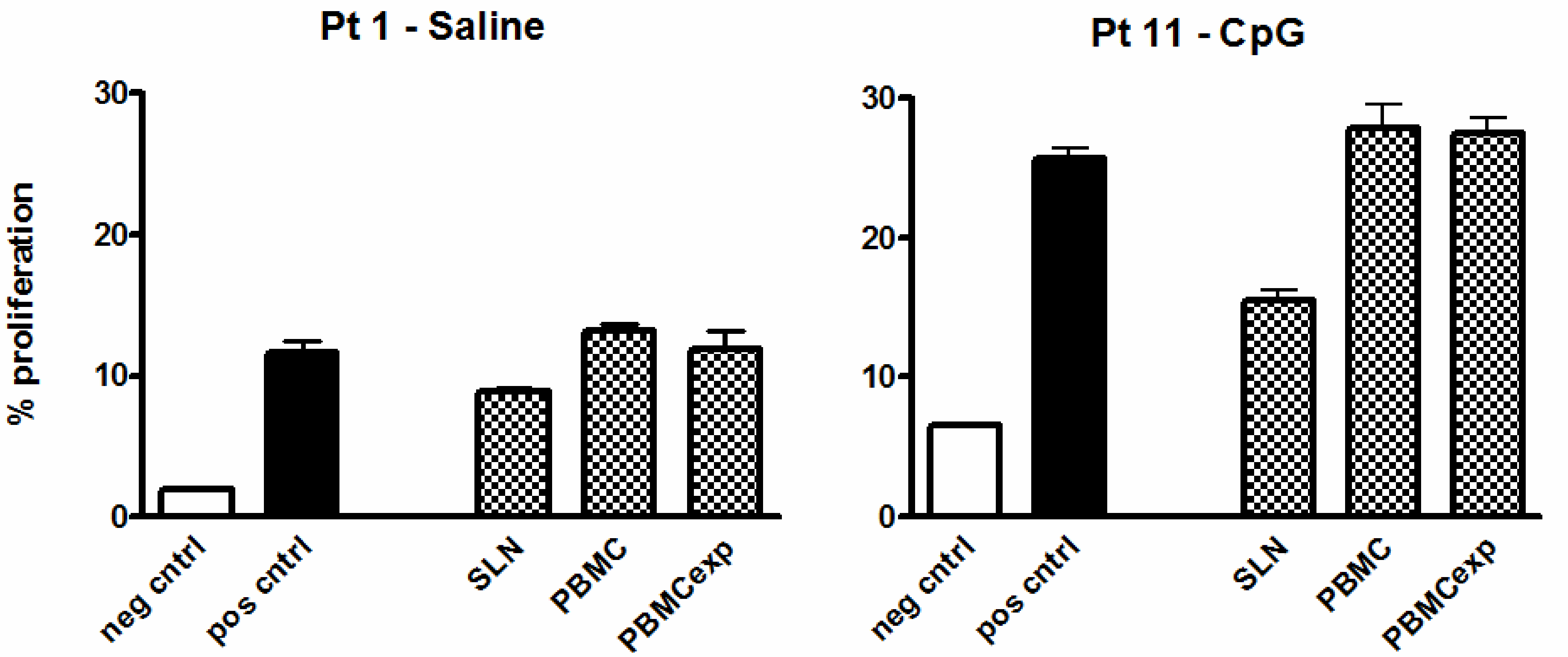

Suppl. Fig. 1. Suppression assays from a saline and a CpG administered patient. Negative control (open bar)=unstimulated CD4+CD25- effector T cells, positive control=stimulated CD4+CD25- effector T cells. Gray bars show the percentage of proliferated CD4+CD25- effector T cells in the presence of CD4+CD25+ enriched fractions of respectively expanded SLN T cells (SLN), peripheral blood T cells (PBMC) and expanded peripheral blood T cells (PBMCexp) harvested at the same time point (t=0).

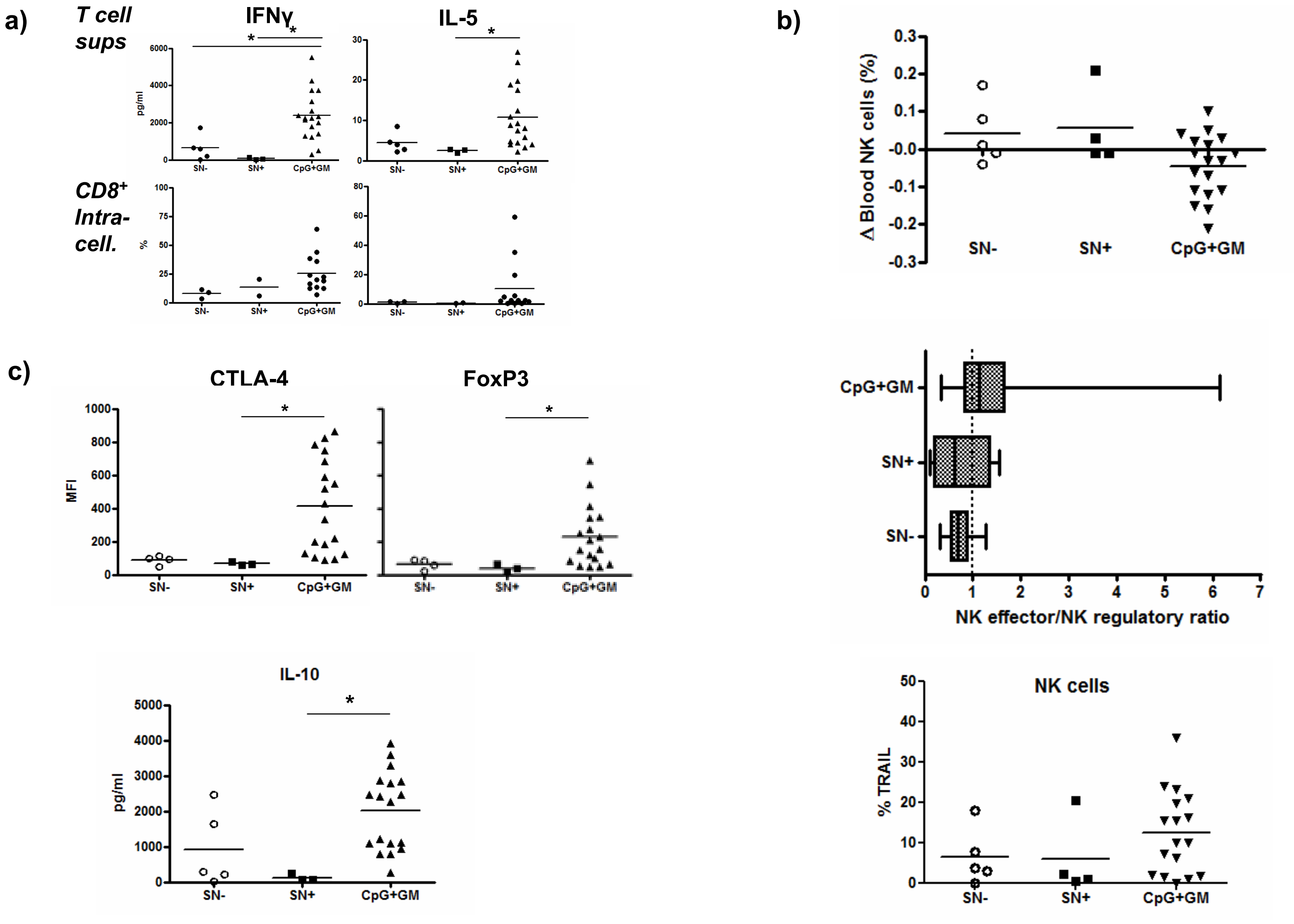

Suppl. Fig. 2 Immune stimulatory and regulatory effects are related to CpG±GM administration and not to the presence of tumor cells a) Representative T cell cytokine profiles after in vitro stimulation stratified by tumor negative or positive saline and CpG±GM administered patients. Means (in bar graphs with SEM) are shown. *P<0.05. **b)** Pre- and post-treatment changes in NK cell frequencies in the peripheral blood, shift from predominantly regulatory $CD56^{bright}$ to more effector $CD56^{dim}$ NK cells in the melanoma SLN, and surface TRAIL expression on SLN NK cells stratified by tumor negative or positive saline and CpG±GM administered patients. *P<0.05. **c)** Mean Fluorescence Intensity (MFI) of CTLA-4 and FoxP3 expression in SLN Tregs and IL-10 secretion after stimulation of SLN T cells stratified by tumor negative or positive saline and CpG±GM administered patients. * P <0.05.
